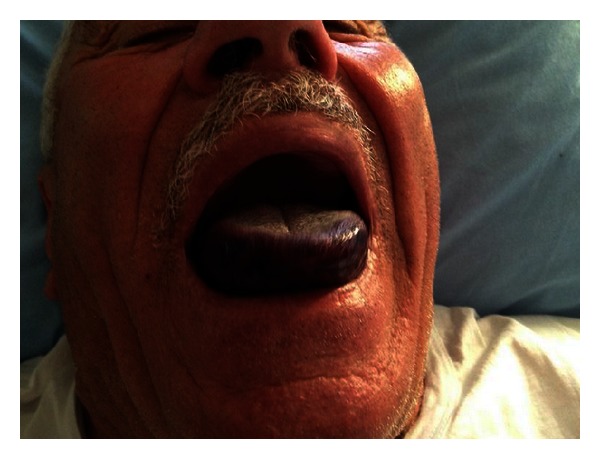# Lingual Haematoma due to Tenecteplase in a Patient with Acute Myocardial Infarction

**DOI:** 10.1155/2013/239796

**Published:** 2013-06-03

**Authors:** Muhlis Bal, Yavuz Atar, Ziya Salturk, Ahmet Hakan Ateş, Serkan Yağcı, Gökçen Coşkun Bal

**Affiliations:** ^1^Sinop Atatürk Government Hospital, Otorhinolaryngology Department, 57000 Sinop, Turkey; ^2^Kaptanpaşa Mahallesi Halit Ziya Türkkan Caddesi, Middleist Blokları A Blok No. 10-12 D:25, Okmeydanı, 34384 İstanbul, Turkey; ^3^Sakarya Yenikent Government Hospital, Otorhinolaryngology Departmant, 54000 Sakarya, Turkey; ^4^Suluova Government Hospital, Otorhinology Departmant, 55000 Suluova, Turkey; ^5^Sinop Atatürk Government Hospital, Pathology Department, 57000 Sinop, Turkey; ^6^Okmeydanı Training and Research Hospital, Otorhinolaryngology Clinic, 34384 İstanbul, Turkey

## Abstract

The use of intravenous thrombolytic agents has revolutionised the treatment of acute myocardial infarction. However, the improvement in mortality rate achieved with these drugs is tempered by the risk of serious bleeding complications, including intracranial haemorrhage. Tenecteplase is a genetically engineered mutant tissue plasminogen activator. Haemorrhagic complications of tissue plasminogen activator (tPA) are well known. Compared to other tPAs, tenecteplase use leads to lower rates of bleeding complications. Here, we report a case of unusual site of spontaneous bleeding, intralingual haematoma during tenecteplase therapy following acute myocardial infarction, which caused significant upper airway obstruction and required tracheotomy to maintain the patient's airway. Clinical dilemmas related to securing the airway or reversing the effects of tissue plasminogen activator are discussed.

## 1. Introduction 

Tissue plasminogen activators (tPAs) are accepted agents for the therapy of selected cases of acute ischaemic cerebrovascular events, such as myocardial infarction, pulmonary embolism, portal vein thrombosis, and deep venous thrombosis [1, 2]. As a result of its nonselective ability to lyse clots throughout the vascular system, complications caused by bleeding involving various organs and organ systems have been reported. Tenecteplase (TNKase; Genetech Inc.) is an engineered variant of tPA (Activase; Genentech Inc.) designed to have increased fibrin specificity, and its greater efficacy leads to lower rates of bleeding complications [3]. Lingual haematoma is a rare but potentially fatal cause of upper airway obstruction. It is important to recognise this unusual clinical entity early in its course and take the appropriate steps to secure the airway [4]. Here, we report a case of acute airway compromise secondary to a lingual haematoma that developed after administration of recombinant tPA for acute myocardial infarction (AMI).

## 2. Case Report

A 73-year-old male patient presented to the emergency department (ED) with severe retrosternal chest pain, which progressed over time. Electrocardiogram revealed ST-segment elevation in leads D2, D3, aVF, and V4–V6 derivations and ST depression at D1, aVL, and V1–V3 derivations. He had a history of well-controlled hypertension and two prior nonhaemorrhagic strokes without residual deficits. The pulse rate was between 80 and 95 beats/min and blood pressure was 130/90 mmHg. Evaluation of the patient's coagulation status showed slight increases in prothrombin time and international normalised ratio (13.9 s and 1.4, resp.). The results of serum blood chemistry studies, blood cell counts, and other coagulation studies were within the respective normal limits. A bolus of recombinant tPA was administered and infusion was started within 2 hours following the onset of symptoms. Aspirin (300 mg), heparin (5000 IU intravenous bolus), and tenecteplase infusion (8000 IU IV bolus over 5–10 s) were administered. After completion of thrombolytic therapy, he complained of right hemiglossal numbness and a swelling in the mouth within 60 min. He consulted our otorhinolaryngology department. Physical examination revealed significant swelling and purple discolouration of the tongue (Figure 1). There was no history of trauma. No signs of airway compromise were observed, but there was difficulty in swallowing saliva, drooling, and coughing and speech disorder due to extensive swelling. To determine the posterior extent of the swelling, we performed fibre optic transnasal endoscopic examination of the upper airway over the next 30 min. The haematoma extended to the glossoepiglottic fold, without involving the epiglottis. The patient was asked not to talk and not to extend the tongue as much as possible to help in immobilisation. The physical and endoscopic examinations were repeated frequently. The size of the lingual haematoma increased and respiratory difficulty occurred. As conventional intubation could not be performed due to not having enough passage; emergent tracheotomy was performed under local anaesthesia. The patient was admitted to the coronary intensive care unit for further monitoring and supportive care. Heparin infusion was discontinued and both protamine and fresh frozen plasma were administered. The lingual haematoma resolved slowly. We continued antiplatelet treatment but cessation of anticoagulant therapy contributed to resolution of the haematoma. Three days after this operation, flexible endoscopic and intraoral examinations revealed that lingual haematoma was completely resolved and therefore the tracheotomy was closed.

## 3. Discussion 

Tenecteplase is a third-generation thrombolytic agent indicated for the reduction of mortality associated with AMI. This is a triple combination mutant variant of alteplase with high fibrin specificity and resistance to plasminogen activator inhibitor 1. The reduced rate of systemic clearance of the drug relative to alteplase enables prolonged half-life, which allows tenecteplase to be given as a single rapid bolus injection to patients with AMI with ST segment elevation [3, 5].

Intraoral haematomas typically result from trauma [6] after motor vehicle accidents [7, 8] or traumatic tracheal intubation in warfarin-treated patients or patients receiving thrombolytic therapy [6]. Other unique bleeding episodes, including pulmonary and ocular haemorrhage, hemopericardium, and haemorrhage into an ovarian cystadenocarcinoma, have been reported [9–11].

It has been reported that the frequency of major bleeding after thrombolytic treatment is 5–7%. Trauma [12] and anticoagulation with heparin, warfarin, streptokinase, and/or tissue plasminogen activator [1] are the most common causes of lingual haematoma. In addition, one case has been attributed to severe hypertension [13]. However, there are insufficient data in the literature regarding the possibility of lingual haematoma.

The most common problem involving intraoral haematomas is their potential to expand progressively and cause acute airway obstruction requiring emergent tracheal intubation, tracheotomy, or cricothyroidotomy [14]. Patients can present with varying degrees of airway compromise, ranging from none at all to complete obstruction of the upper airway. To date, 17 cases of lingual haematoma have been described in the literature; five were surgically managed with tracheotomy and six were managed with orotracheal or nasotracheal intubation; the remaining six patients were kept under expectant observation [15].

Initially, it is important to assess, and if necessary secure, the airway. In all cases, a sore throat seemed to be an early symptom, followed by dysphagia, hoarseness, drooling, or respiratory distress [6]. Surgical drainage of the haematoma is generally not indicated.

In our case, repeated flexible nasal endoscopies were performed to determine whether surgical management would be required. As a result of progression, we decided to perform tracheotomy. The decision regarding whether such an intervention is required or not should always be based on the clinical presentation. If any indication of severe obstruction or oedema within the pharynx or larynx is detected by endoscopic examination, a surgical airway should be established immediately. Tracheotomy under local anaesthesia was the most appropriate course of action in our case. Indeed, the potential for lingual haematoma to completely obstruct the upper airway must not be overlooked.

We concluded that oropharyngeal examination is important after administration of thrombolytic therapy. Identification of this potentially life-threatening complication, as well as continued attention to the ABCs (airway, breathing, and circulation) during and after administration of the thrombolytic agent, enables rapid airway management in a controlled manner.

## Figures and Tables

**Figure 1:**